# Psychopathological Functioning Levels (PFLs) and their possible relevance in psychiatric treatments: a qualitative research project

**DOI:** 10.1186/s12888-016-0940-4

**Published:** 2016-07-20

**Authors:** Andrea Ferrero, Barbara Simonelli, Simona Fassina, Elisabetta Cairo, Giovanni Abbate-Daga, Enrica Marzola, Secondo Fassino

**Affiliations:** Psychotherapy Unit, Mental Health Department, Health District TO-4, Settimo Torinese Turin, Italy; Eating Disorders Center for Treatment and Research, Department of Neuroscience, University of Turin, Via Cherasco 15, 10126 Turin, Italy; SAIGA Institute for Research, Turin, Italy

**Keywords:** Psychopathology, Psychopathological functioning, Personality organization

## Abstract

**Background:**

Symptoms description is often not enough to provide clinicians with guidelines for treatments and patients’ clinical history does not represent an exhaustive source of data. Psychopathological dysfunctions are known to relate to the core disturbances that underlie different forms of psychopathology so the identification of such dysfunctions could be helpful for treatments. Some tools are available although highly complex and lengthy. This study aimed to provide clinicians with an easy-to-administer instrument able to capture different levels of impairment in psychopathological functioning, namely the Psychopathological Functioning Levels – Rating Scale (PFL-RS).

**Methods:**

The Psychopathological Functioning Level - Research and Training Committee (PFL-RTC) has been established in Turin since 2002 including psychiatrists and clinical psychologists with extensive clinical and research experience. Our research was grounded on the Qualitative Research Criteria (QRC) 1-7 and conducted with subsequent steps in order to identify those core psychopathological dysfunctions to be rated by this tool.

**Results:**

From 2002 until 2014, 316 outpatients were administered the clinical interview on at least two different occasions. Diagnoses were mixed and included: Schizophrenic and Psychotic Disorders, Depressive Disorders, Anxiety Disorders, Obsessive-Compulsive Disorder, Post- Traumatic Stress Disorder, Somatic Symptoms Disorders, Eating Disorders and Personality Disorders. Focus groups were conducted to identify those psychopathological dysfunctions which needed to be rated, according to two Phenomenological Selection Criteria (PhSC) and four Etiopathogenetic Selection Criteria (EtSC). As a result, five dysfunctional areas emerged: Identity (ID); Comprehension (CO); Negative Emotions (NE); Action-Regulation (AR); Social Skills (SS). After checking such dimensions for consistency with the existing instruments, 7 levels of severity were identified for each area. Finally, a provisional Italian schedule of Psychopathological Functioning Levels – Rating Scale (PFL-RS) was obtained and checked for semantic comprehension and then administered gathering preliminary data.

**Conclusions:**

Psychopathological dysfunctions underlying mental disorders have been recognized in the present study with the PFL-RS. This instrument seems promising to inform in a specific way treatments strategies and goals, specifically concerning psychotherapy. Notwithstanding, further research is needed in order to confirm validity, sensitivity and reliability of this instrument.

## Background

According to literature, symptoms description is not suitable to provide clinicians with guidelines for treatments [1, 2]; similarly, patients’ clinical history does not represent an exhaustive source of data able to guide therapists’ reasoning [3].

Evidence showed that the comprehension of many psychic disorders can be increased by the identification not only of primary psychopathological dysfunctions underpinning each disorder [4–6], but also of those factors affecting their development [7, 8].

To date, psychopathological dysfunctions are known to relate to core disturbances underlying different forms of psychopathology [9, 10]. Therefore, the use of specific encoded features [11] to identify such dysfunctions could help plan treatments [12] and provide clinicians with individualized treatment options for their patients.

According to these novel lines of research, some recent diagnostic manuals, mainly psychodynamically oriented, like the Shedler Westen Assessment Procedure – 200 (SWAP-200) [13], the Operationalized Psychodynamic Diagnostics - 2 (OPD-2) – Axis 4 [2], and the Psychodynamic Diagnostic Manual (PDM) – Axis M [6], focused on correlating patients’ idiographic and nomothetic aspects in order to describe different levels of psychic organization, according to homogeneous clusters of variance.

Unfortunately, the aforementioned instruments are highly complex and need further validation.

With more detail, some OPD dimensions [2] overlap thus are strictly interdependent; moreover, they are not enough sensitive when measuring patients’ changes (i.e., Conflict Axis and Structure Axis). Similarly, some PDM Axis M categories [6] often overlap, since they aim to outline infinitely complex processes. Moreover, certain terms resulted to be either unfamiliar or relying excessively on a particular theoretical jargon [2, 14]. So, greater detail and specificity in describing patients’ psychopathology could be only pursued depending on time and experience [14].

Otherwise, the Five Factor Model (FFM) focuses on the variation of psychopathology at the adaptive poles, without explicitly measuring features located at the maladaptive ones [15].

Furthermore, the DSM-IV-TR Axis V- Global Assessment of Functioning (GAF) Scale does not have sufficient specificity to be useful in measuring severity of psychopathological dysfunctions [14].

Finally, the proposed criteria of the Appendix - Section 3 of the DSM-5 [16] try to represent in a novel way core psychopathological impairments in psychic functioning dimensions. However, they are proposed as crucial only with respect to personality disorders. Nevertheless, such criteria to date need further support and seem too complex to be successfully applied to every-day clinical work [17].

The overarching aim of this study was to provide clinicians with an easy-to-administer instrument able to capture different levels of impairment severity in psychopathological functioning, namely the Psychopathological Functioning Levels – Rating Scale (PFL-RS). This tool is characterized by the following features:

1. flexible use in order to maximize clinical utility;
2. clear expression of clinical and diagnostic guidelines in order to allow clinical assessments [2];
3. use of an intermediate level of abstraction between behavior description and meta-psychological concepts [2];
4. synthesis of the various concepts across self-other models to form a foundation for rating psychopathological functioning;
5. high formalization: an accurate description of those characteristics corresponding to each item is preferable when measuring the impairment in psychic functioning;
6. accessibility of language for clinical assessment [2];
7. ease of administration: this tool is not designed to be used only by researchers with extensive training.

To our knowledge, no existing model and related assessment instrument encompasses this complete set of characteristics.

## Methods

The Psychopathological Functioning Level - Research and Training Committee (PFL-RTC) has been established in Turin since 2002 as a part of the Adlerian Psychodynamic Psychotherapies - Training and Research (APPs - TR) network, including: a) the Eating Disorders Program of the University of Turin, Italy; b) the Società Adleriana Italiana Gruppi e Analisi School of Psychotherapy in Turin, Italy; and c) the Department of Mental Health, Psychotherapy Unit - ASL Turin 4, Italy.

This network includes: scientific promoter and coordinator (Andrea Ferrero), scientific supervisor (Secondo Fassino), scientific secretaries (Barbara Simonelli, Simona Fassina, Elisabetta Cairo, Giovanni Abbate-Daga), and researchers (psychiatrists, psychologists and psychotherapists).

### Research theoretical background

Qualitative research aims to broaden the understanding of clinical experiences and phenomena as they are lived through situations, rather than testing hypothesized relationships or causal explanations, that benefit best from quantitative methods [18, 19]. This central purpose is common to different approaches that have been developed in qualitative research. Nevertheless, each of them have its own traditions and methods, and a specification of the researchers’ conceptual frameworks is therefore required [18, 19] and guidelines have been developed in this regard [20].

In order to conveniently assess and present the results of our research, the following Qualitative Research Criteria (QRC) have been taken into account:

- (QRC1) the range of persons and situations to which the findings might be relevant are described;
- (QRC2) authors specify their theoretical orientations;
- (QRC3) coherence of the understanding with underlying structure for the phenomenon or domain is explained;
- (QRC4) the way of accomplishing specific research task is described;
- (QRC5) credibility checks (comparing the results with two or more varied qualitative perspectives) are provided;
- (QRC6) reviewers’ judgements are provided;
- (QRC7) some brief clinical examples are proposed.

They were adopted with reference to the “Evolving guidelines for publication of qualitative research studies in psychology and related fields” – Table 1/B: “Publishability Guidelines Especially Pertinent to Qualitative Research” [19]. In more detail, QRC1 refers to the criterion B2 of the aforementioned Guidelines, QRC2 to the criterion B1, QRC3 to the criterion B5, QRC4 to the criterion B6, QRC5 to the criterion B4, QRC6 to the criterion B7, and QRC7 to the criterion B3. Finally, also the Consolidated Criteria for Reporting Qualitative Research (COREQ) [20] checklist has been applied.

### Project steps

In line with the procedures used by OPD-2 and DSM-5 [2, 16], subsequent steps were taken to identify core psychopathological dysfunctions to be rated by the novel instrument and to refine the levels of impairment in order to be diagnosed by a specific rating scale.

- (Step A) Describing subjects of investigation (QRC 1) with multiple research meetings aiming to ascertain the feasibility of the study also discussing which diagnoses could be eligible;
- (Step B) Defining clinical domain and psychopathological theoretical model (according to QRC 1-2) specifying the psychopathology areas that the Authors aimed to refer to (QRC2) referring to the Vulnerability Events Personality - Psychopathological Model (VEP-PM);
- (Step C) Identification of core psychopathological dysfunctions (according to QRC 3-4) with a detailed analysis of the literature on such aspects;
- (Step D) Consistency with current research of the “Five psychopathological dysfunctions - rating model” (according to QRC 5) with respect to neuroimaging studies, Psychodynamic Diagnostic Manual – Axis M (PDM-M) 16 items [6] and Operationalized psychodynamic – Axis 4 (OPD-4) 13 items [2], “Personality Functioning Levels” and “Psychopathological traits” according to DSM-5 Appendix – Section 3 [16];
- (Step E) Identification of different rating levels of psychopathological dysfunctions (according to QRC 3-4) with multiple 90-minute focus groups on this topic;
- (Step F) Consistency of the “Seven impairment levels - rating model” with current research (according to QRC 5) through a deep understanding and evaluation of timely literature carried out by the PFL-RTC;
- (Step G) Providing a provisional Italian schedule of Psychopathological Functioning Levels – Rating Scale (PFL-RS) based on earlier work of the group to describe different impairment levels in the selected psychopathological areas;
- (Step H) Checking semantic comprehension of PFL-RS definitions by Mental Health professionals (according to QRC 6) during weekly research meetings of the PFL-RTC;
- (Step I) Psychopathological Functioning Levels – Rating Scale (PFL-RS): Italian and English schedules; translation and back-translation were carried out by native-speaker psychiatrist and psychotherapist;
- (Step J) Preliminary data on the administration of the PFL-RS instrument to a clinical sample (according to QRC 7) were obtained by the Committee interviewing 54 outpatients of a Psychotherapy Unit in Settimo Torinese, Turin, Italy.

## Results

### (Step A) Clinical sample general characteristics

From 2002 until 2014, 316 outpatients referring to the Department of Mental Health - ASL Turin 4, Italy, were administered semi-structured clinical interviews on at least two different occasions before starting treatments. Participants were 84 males (26.5 %) and 232 females (73.5 %) with a mean age of 37.9 ± 11.9 years. To be included in this study, patients had to be diagnosed with the following diagnoses (according to the DSM-5 [16]): Schizophrenic and Psychotic Disorders, Depressive Disorders, Anxiety Disorders, Obsessive-Compulsive Disorder, Post-Traumatic Stress Disorder, Somatic Symptoms Disorders, Eating Disorders and Personality Disorders. Six individuals (1.9 %) were diagnosed with Schizophrenic and Psychotic Disorders, 63 (20 %) with Depressive Disorders, 129 (40.8 %) with Anxiety Disorders, 13 (4.1 %) with Obsessive-Compulsive Disorder, 7 (2.3 %) Post-Traumatic Stress Disorder, 13 (4.1 %) Somatic Symptoms Disorders, 11 (3.4 %) Eating Disorders and 74 (23.4 %) with Personality Disorders.

### (Step B) Definition of clinical domain and psychopathological theoretical model of the research

The tool to be developed is intended to assess different levels of psychopathological functioning underlying a variety of psychiatric disorders (QRC1). To this end, it must be specified which psychopathology the Authors refer to (QRC2).

In order to fulfil this requirement, the PFL research specifically referred to the Vulnerability Events Personality - Psychopathological Model (VEP-PM), a specific bio-psycho-social model [21–24] for the comprehension and treatment of psychiatric disorders. VEP-PM is rooted in phenomenology, psychodynamics, and neuroscience [25–35]. More in detail, VEP-PM is grounded on Alfred Adler’s psychodynamic psychopathology [25, 36], which is consistent with Cloninger’s definition [37] of temperament and character dimensions and current approaches to psychopathology thus highlighting the developmental perspective in the understanding of the functional structures of the mind [38].

According to the VEP-PM, the following pathogenic factors are considered:

- biological and psychosocial vulnerability;
- relevance of significant life events;
- Personality Organization.

Factors a) and b) are combined to generate suffering (VEP-PM causative psychopathological factors), while factor (c) rises from the patient’s attempt to face it (VEP-PM compensatory psychopathological factor).

### (Step C) Identification of core psychopathological dysfunctions

A focus group of 8 experienced clinicians and researchers (3 males and 5 females) working at the APPs-TR network (3 psychiatrists and 5 clinical psychologists) discussed and proposed criteria to identify those psychopathological dysfunctions which needed to be rated, also considering those targets potentially helpful in managing treatments across a wide range of psychic disorders. Also, data saturation was discussed.

Two Phenomenological Selection Criteria (PhSC) and four Etiopathogenetic Selection Criteria (EtSC) were chosen to highlight significant specific dysfunctional areas.

#### Phenomenological selection criteria (PhSC)

- (PhSC1) The dysfunctional area relates to phenomenological subjective dimension of experiences.
- (PhSC2) The dysfunctional area relates to phenomenic objective dimension of behaviours.

#### Etiopathogenetic selection criteria (EtSC)

- (EtSC1) The dysfunctional area relates to empirical studies of disordered personalities.
- (EtSC2) The dysfunctional area deals with intrapsychic and relational aspects.
- (EtSC3) The dysfunctional area includes both verbally and non-verbally expressed attitudes.
- (EtSC4) The dysfunctional area relates to vulnerability and subjective relevance of significant life events (“VEP-PM causative psychopathological factors”).

As a result, five dysfunctional areas finally met the aforementioned required criteria.

##### Identity (ID)

This area refers to self and others’ representations [39, 40], and includes 2 sub-dimensions.

1. Identity differentiation and constancy of self and others’ representations.
  Indicators of this dimension involve important functions, e.g. identity integration vs. fused or confused self-other representations [2, 6, 38, 41, 42].
2. Anti-ambivalent or hyper-ambivalent identity.
  Anti-ambivalent (precarious) identity means that contradictory aspects of self and others’ representations persist in a disconnected way [2, 39]. Hyper-ambivalent identity represents constancy, cohesion, and sense of relatedness which are preserved in an oscillatory and ambivalent way [2, 39].

##### Comprehension (CO)

It refers to “Verstehen” unlike “Erklären” phenomenological dimension [35]. This area includes 3 sub-dimensions.

1. Ability to differentiate and integrate thoughts.
  It is impaired by reduced empathy, and intrapsychic [6] or environmental stressors [2]. Impairment may result in fragmentation, polarized divergences, concrete and somatic representations [6, 41].
2. Reflection ability (e.g., executive functioning, attention and signals elaboration).
  It is impaired because of a conflicting over regulation [2] resulting in oversimplification, displacement, control of causes, and avoidance of effects [6].
3. Planning ability.
  The latter deals with evaluation of different values, objectives, expectations or contexts, with prevision of environmental reactions, with creativity.

##### Negative emotions (NE)

It refers to relational hardwired emotional connections [43], and includes 3 sub-dimensions.

1. Negative core (basic) emotional feelings.
  This first sub-dimension is represented by fear, rage and panic, as brain’s systems that generate instinctual-emotional behaviors [44, 45]. They are necessary but not sufficient to generate higher order emotional expressions.
2. The lack of positive basic emotional feelings.
  They moderate negative basic emotions.
3. Higher order negative emotional feelings.
  They include shame, guilt, jealousy, envy, distrust, anxiety, and sadness [46] that derive from the interaction of the basic emotional system with learning (e.g., higher cognitive processes and culture).

##### Action-regulation (AR)

It refers to behaviors and impulse control dysregulations [47]. This area includes 3 sub-dimensions.

1. Dysregulated behaviors.
  Solipsististic and social retirement, as well as harmful and damaging behaviours toward one’s self and others [41] may be intentionally or impulsively acted [2], threatened or imagined.
2. Maladaptive behaviors.
  They result from the inadequate evaluation of environmental requirements under regulation and integration [2], reduced prevision and reward delay [2], and inappropriate focusing [6].
3. Restrained behaviours.
  They result from alexithymia, inhibition, or hyper regulation [2] relating to excessive reward dependence or markedly self-centered novelty seeking [6].

##### Social skills (SS)

It refers to relationships, epistemic trust and culture [48] and includes 3 sub-dimensions.

1. Basic social autonomy (self-care, nutrition, dwell, and rules of living together). This sub-dimension deals with proper or emotionally disturbed perception of a wide range of signals [6].
2. Ability to participate in socio-relational projects.
  It deals with life tasks: love, friendship, study, work, and leisure activities involving loss and satisfaction, attention and distance, flexibility and persistence [2, 6].
3. Ability to promote socio-relational projects
  This aspect determines the aforementioned life tasks but it involves also desire and investment, organization and determination [2, 6], choices and ethics [41].

### (Step D) Consistency with current research of the “Five psychopathological dysfunctions - rating model”

As a subsequent step, the consistency of the five-areas rating model with other models proposed in literature to evaluate specific dysfunctional aspects has been checked.

The identification of the aforementioned core psychopathological areas emerges as positively related to a number of current research findings.

1. Neuroimaging studies demonstrate that brain is not only active while when stimulated, but also during the resting state. Therefore, resting state’s spatiotemporal structure is central and may serve as the neural predisposition of what psychodynamics describe as the psychological structure of the Self, interacting with several brain processes relating to sensorimotor, affective, cognitive and social domains [49]. Cognitive, affective and social neurosciences thus justify a dimensional approach to psychopathological dysfunctions focusing Identity (ID), which specifically refers to the resting-state intrinsic activity of the brain, as well as Cognition (CO), Negative Emotions (NE), Action-Reaction (AR) and Social Skills (SS), as dimensions which refer to specific levels of bottom-up processes of brain activity faced to extrinsic situations [49].
2. Psychodynamic Diagnostic Manual – Axis M (PDM-M) 16 items [6] and Operationalized psychodynamic – Axis 4 (OPD-4) 13 items [2] are specifically devoted to the assessment of mental functioning and structure. Comprehensively, all the five selected areas of this model are considered by both manuals, and in turn there are no PDM and OPD items which are not included.
  Identity (ID) differentiation and constancy of self and others’ representations, as well as the characteristics of anti-ambivalent or hyper-ambivalent identity are investigated in PDM-M Self-Observation, Internal Representations, and Defensive Patterns subscales rather than in OPD-4 Self-Perception and Defences subscales.
  Capacity of differentiating and integrate thoughts, reflection (executive functioning, attention, and signals elaboration) and planning, altogether refer to the Comprehension (CO) area of this model, and are described in PDM-M Self-Observation, Internal Representations, Differentiation-Integration and Regulation subscales [6], rather than in OPD-4 Self-Perception and Self-Regulation subscales [2].
  Negative Emotions (NE), namely negative core (basic) emotional feelings, lack of positive basic emotional feelings and higher order negative emotional feelings, are included in PDM-M Internal Experience and Affective-Communicative Experiences subscales [6], as well as in OPD-4 Self-Perception subscale [2].
  Dysregulated, maladaptive and restrained behaviours, as components of the Action-Regulation (AR) area, are assessed in PDM-M Internal Representations, Regulation, and Moral Sense subscales [6] and in OPD-4 Self-Regulation and Communication subscales [2].
  Finally, Social Skills (SS), including basic social autonomy, and the ability to access socio-relational projects and to promote socio-relational projects, are detected in PDM-M Self-Observation, Differentiation-Integration, Affective-Communicative Experiences, Relationships-Intimacy, Regulation and Moral Sense subscales [6]; otherwise they are included in OPD-4 Self-Regulation, Object Perception, Bond and Communication subscales [2].
3. Furthermore, this model has been also found to assemble in a different way both “Personality Functioning Levels” and “Psychopathological traits” according to DSM-5 Appendix – Section 3 [16]. More in detail, Identity (ID) was partially consistent with DSM-5 item “Identity” and “Psychoticism” [16]; Comprehension (CO) variously refers to DSM-5 items “Identity”, “Self-Direction”, “Empathy”, and “Psychoticism” [16]. Relatedly, Negative Emotions (NE) deal with DSM-5 items “Identity”, “Emotional lability”, “Anxiousness”, “Separation insecurity”, “Depressivity”, and “Hostility” [16]; Action-Regulation (AR) pertains to DSM-5 items “Impulsivity”, “Risk taking”, and “Compulsivity” [16]. Finally, Social Skills (SS) relate to DSM-5 items “Intimacy”, “Separation insecurity”, and “Hostility” [16].
4. Different types of patients may be differentially responsive to different forms of treatments and discrete factors serve as moderators, mediators, and proxies for therapeutic change [50]. Among those that have been already highlighted [44], representations of one’s self and others are consistent with Identity (ID), Comprehension (CO), Negative Emotions (NE), and Social Skills (SS) areas, while capacities for reality testing, mentalization, and reflective functioning are consistent with the Comprehension (CO) area; finally, the background of safety or perceived security is consistent with Comprehension (CO), Negative Emotions (NE), Action-Regulation (AR), and Social Skills (SS) areas.

### (Step E) Identification of different rating levels of psychopathological dysfunctions

Experts’ consensus has been reached on the evaluation of severity in order to create a solid dimensional system of psychopathology [51]. According to recent research [52], there is a correlation between both severity and disability of a mental disorder and the level of impairment of individuals’ psychopathological functioning. Relatedly, outcome is predicted in a more accurate way by severity than by a mere disorder classification [17, 53].

More specifically, prior research with reference to “primitive”, “intermediate”, and “more advanced” levels of dysfunctions that characterize anaclitic and self-definitional disorders indicated that patients’ response to treatment is consistent with their diverging Personality Organization [54].

According to the VEP-PM theoretical model, the APPs-TR network focus-group decided that different dysfunctional levels in these areas should consider Personality Organization in terms of mechanisms of defence, coping, and creative compensations. These mechanisms are overall considered to underlie the quality of patient’s attempts to face and reduce pain and discomfort.

Different dysfunctional degrees in core psychopathological dysfunctions were therefore considered only if meeting the following Etiopathogenetic Selection Criterion (EtSC).

(EtSC5) The levels of psychopathological dysfunctions relate to Personality Organization (“VEP PM compensatory psychopathological factor”), that is each impairment level should correspond to a different Personality Organization (PO).

PO refers to a set of enduring, mostly unconscious, brain mechanisms [12, 55] and psychological structures that dynamically organize mental processes and contents into a coherent organization. These dynamically organized structures and processes are assumed to be involved in self-structure, cognition, affect regulation, impulses, and quality of relationships [40, 56, 57], in turn determining the levels of impairment.

Nevertheless, PO is still a latent construct, which can only be inferred from manifest indicators [57].

Seven Psychopathological Functioning Levels (PFL) of each area met the required selection criterion.

They were ordered progressively [58] along a continuum [17] of pathological PO [59, 60], according to Kernberg’s model [61] and subsequent integrations provided by recent research focusing on defence mechanisms, sharing the assumption that levels of PO follow a developmental progression from severely undifferentiated and disorganized levels of PO to mature, integrated, and differentiated levels of PO [12].

In fact, the Borderline Personality Organization, which was originally divided in two levels [61], was classified in this study into three levels, according to prototypical descriptions of the Structured Interview of Personality Organization (STIPO) [Clarkin JF, Caligor E, Stern BL, Kernberg OF. Structured Interview of Personality Organization (STIPO). New York, unpublished document 2004] and to the Defence Mechanisms Rating Scale (DMRS) findings [62]: 1) with prevalent use of major image-distorting defences, 2) with prevalent use of minor image-distorting defences, 3) with prevalent use of both minor image-distorting and disawoval defences.

Additionally, a specific level was dedicated to the coexistence of Neurotic and Borderline Personality Organizations, namely when the use of the above mentioned immature defences is displayed only in situations characterized by severe threats and/or when it is widely intertwined with the use of neurotic defences (e.g., repression). In addition, this level significantly correlates with “Neurotic 2” PO, according to the STIPO [Clarkin JF, Caligor E, Stern BL, Kernberg OF. Structured Interview of Personality Organization (STIPO). New York, unpublished document 2004].

- ❖ *Level 1* refers to Psychotic Personality Organization;
- ❖ *Levels 2, 3, and 4* refer to Borderline Personality Organizations;
- ❖ *Level 5* refers to coexistence of Neurotic and Borderline Personality Organizations;
- ❖ *Level 6* refers to Neurotic Personality Organization;
- ❖ *Level 7* refers to mature Personality Organization.

### (Step F) Consistency of the “seven-impairment levels” rating model with current research

This seven-impairment levels rating model emerges as positively related to a number of current research findings.

1. According with more recent views of personality pathology as developmental delays as opposed to failures [63, 64], PO is not conceived as a rigid (adaptive or maladaptive) structure, but as a flexible set of psychic dynamics that may vary along the lifespan, proceeding from early childhood interactions [65–67] and depending also from significant life events. Therefore, there is a correlation of PO with more stable character traits and more extemporaneous responses to state of mind [68].
  PO not only describes different levels of defences, as they were ranked by several rating scales (DSM IV – TR Defense Functioning Scale-DFS [69], Defense Style Questionnaire – DSQ [70], Defense Mechanisms Rating Scales – DMRS [71]), but also the degree of creative exploration and mental transformation of experiences [42]; moreover, dysfunctional defences might be adaptive in certain circumstances [68].
2. Defences may make dysfunctional areas resistant to change [62]. To provide a few examples, splitting defences may hinder self-cohesion, pertaining to Identity (ID); denial or repression may interfere with cognitive processes and beliefs, pertaining to Cognition (CO); introjection or affect isolation can relate to persistence of emotionally significant experiences (e.g., rage or feeling of emptiness), pertaining to Negative Emotions (NE); acting-out affects surface traits (e.g., impulsiveness or inhibition), individual quality of relationships, and specific behaviors (e.g., self-mutilation or attempt of suicide), that pertain to Action-Regulation (AR) and Social Skills (SS). These findings constitute an additional reason for distinguishing different levels of dysfunctional areas with reference to individuals’ PO.
3. Furthermore, recent studies demonstrate [72] that PO has not only to be considered as a set of processes employed against particular impulses or wishes, but it may be seen as developing within the context of close relationships with relevant others, and may serve as a means for satisfying socio-relational goals [36, 73]. Thus, PO can be reasonably also valued as a modulating factor of interpersonal style and coping, pertaining to the Social Skills (SS) area, behaviours dysregulation, pertaining to Action-Regulation (AR), and social cognition, pertaining to Cognition (CO). For example, idealization or projection mechanisms influence relationships.
4. From a clinical point of view, a significant differentiation of personality disorders, depression, and anxiety groups by defence use alone was repeatedly found [74, 75].
5. Pretreatment PO characteristics were found to be predictors of sustained therapeutic change [54]. A recent systematic review [12] suggests that higher initial levels of PO are moderately to strongly associated to better treatment outcome, and different levels of PO may interact with the type of psychotherapeutic intervention (e.g., interpretative vs. supportive). Defence style was found to be correlated with both the course of early phases and premature termination of psychotherapy [68] and to influence therapeutic alliance [76, 77]. Finally, defence style can be seen as an outcome measure rather than a predictor [68] and PO could also lead the clinician to appreciate, by repeated measures, if patients’ maladaptive attitudes will change over time towards a better or worse psychic functioning.

### (Step G) Providing a provisional Italian schedule of psychopathological functioning levels – rating scale (PFL-RS)

Finally, the schedule was based on the following steps.

1. “Primary Psychotherapeutic Focus Levels” (PPFLs): the first attempt to describe different impairment levels in the selected psychopathological areas consisted of short lists of patients’ characteristics with the aim to individualize treatments, particularly psychotherapy [78].
2. “Personality Functioning Levels” (PFLs): more comprehensive characterizations of five different dysfunctional levels in the selected areas were subsequently recognized, specifically concerning patients with severe personality disorders [22, 79, 80].
3. Psychopathological Functioning Levels – Rating Scale” (PFL-RS): two definitions were provided for seven different levels of psychopathological dysfunctions in the five selected areas, considering both individuals’ internal (self) – and external (others’) perspective.

To date, a number of reliable and valid measures that assess personality functioning and psychopathology demonstrate that a self-other dimensional perspective has an empirical basis and significant clinical utility [81]. This approach was found to be informative in understanding those mental processes associated with psychopathology phenomenology, in planning treatment interventions, in anticipating treatment course and outcome, and in measuring change in treatment [81]. More in detail, current diagnostic and statistical research leads to consider that different degrees of maladaptive integration and balance of interpersonal relatedness and self-definition are involved across a variety of psychopathological pathways underlying symptoms [38]. These recent findings are also consistent with VEP-PM, strengthening Alfred Adler’s assumption concerning the related relevance of social interest and striving for power as effective factors of psychic health or disease. The patient’s emphasis on self-definition (internal perspective) or on relatedness (external perspective) delineates individual’s identity, meanings, and forms of cognition, feelings and affects, behaviors and reactions, qualities of relationships [38].

Patients with psychotic, borderline, neurotic, and mature Personality Organization were included and the PFL-RS provisional schedule overall provided 14 definitions for each area, in a two by two order on a progressive scale.

### (Step H) Checking semantic comprehension of PFL-RS definitions by mental health professionals

The semantic comprehension of the PFL-RS provisional schedule was refined by asking clinicians to read and provide feedback on the proposed definitions of the psychopathological dysfunctions.

All 70 definitions making up the schedule were evaluated for their comprehensibility by 20 reviewers (7 males and 13 females): 5 expert clinicians (2 psychiatrists and 3 clinical psychologists), 10 mental health professionals (MHP) (3 psychiatrists, 2 childhood and adolescence psychiatrists, 2 clinical psychologists, 3 nurses) and 5 trainee graduate psychologists. The task was carried out in two subsequent stages.

After the first reading, 20 corrections have been proposed by reviewers concerning ID area, 13 concerning CO area, 15 concerning NE area, 13 concerning AR area, and 5 concerning SS area. Furthermore, 7 of overall corrections were referring to dysfunctional level 1, 5 to dysfunctional level 2, 3 to dysfunctional level 3, 4 to dysfunctional level 4, 11 to dysfunctional level 5, 10 to dysfunctional level 6, and 1 to dysfunctional level 7.

These corrections were recognized as relevant and accepted by PFL-RTC scientific coordinator, supervisor and secretaries.

A second reading of the derivative revised version of the schedule was provided by reviewers. They added further corrections: 2 were concerning ID area, 1 was concerning CO area, 2 were concerning NE area, 4 were concerning SS area since no additional correction were proposed concerning AR area. Concerning dysfunctional levels, 1 correction was concerning level 3, 7 were concerning level 4, and 1 was concerning level 6, while no additional correction were proposed concerning levels 1,2 and 5. Also these corrections were afterwards implemented into the schedule.

For further details see Table 1.

**Table 1:** Details of the steps needed to achieve the semantic comprehension of the Psychopathological Functioning Levels – Rating Scale (PFL-RS) definitions by Mental Health professionals

Psychopathological Areas	Number of revisions					
	Internal perspective			External perspective		
	1^st^ revision	2^nd^ revision	Total	1^st^ revision	2^nd^ revision	Total
Identity	12	1	13	8	1	9
Comprehension	7	0	7	6	1	7
Negative emotions	7	1	8	8	1	9
Action- regulation	6	0	6	7	0	7
Social relationships	2	3	5	3	1	4
Dysfunctioning levels	Number of revisions					
	1^st^ revision		2^nd^ revision		Total	
Level 1	7		0		7	
Level 2	5		0		5	
Level 3	3		1		4	
Level 4	4		7		11	
Level 5	11		0		11	
Level 6	10		1		11	
Level 7	1		0		1	

### (Step I) Psychopathological functioning levels – rating scale (PFL-RS): Italian and english schedules

As shown in Tables 2, 3, 4, 5 and 6, when PFL-RS definitions were considered satisfactory, the Italian schedule of Psychopathological Functioning Levels – Rating Scale (PFL-RS) was finalized [82]. Subsequently, an English translation of PFL-RS schedule was provided by a mother-language psychiatrist and psychotherapist; a back translation was then conducted to confirm the accuracy of the original text (Tables 7, 8, 9, 10 and 11). Patients were administered the Italian version of the PFL-RS as shown in Tables 2, 3, 4, 5 and 6.

**Table 2:** Psychopathological Functioning Levels – Rating Scale (PFL-RS): Italian schedule

IDENTITA’			
	*Prospettiva interna*		*Prospettiva esterna*
IDi-1	Il soggetto asserisce di percepire la compresenza di altre entità dentro di sé	IDe-1	Il soggetto è convinto che, in alcuni casi, le persone hanno il potere di espropriare gli altri della propria identità (e/o) libertà interiore
IDi-2	L’auto-rappresentazione del soggetto coincide con alcuni suoi atteggiamenti temporanei, senza consapevolezza critica di altri atteggiamenti di segno opposto	IDe-2	Le rappresentazioni che il soggetto si fa dei comportamenti degli altri paiono dettate da un’urgenza occasionale e sono scollegate rispetto ad altre precedenti
IDi-3	Per auto-rappresentarsi in toto, il soggetto utilizza un’immagine settoriale di sé, vissuta alternativamente come tutta positiva (o) tutta negativa	IDe-3	Per farsi un’idea degli altri (e/o) delle relazioni, il soggetto utilizza un’immagine settoriale, alternativamente del tutto positiva (o) del tutto negativa
IDi-4	Di fronte a propri aspetti contradditori, il soggetto ne minimizza (o) disconosce la rilevanza	IDe-4	Di fronte a significati contradditori delle situazioni con gli altri, il soggetto ne minimizza (o) disconosce la rilevanza
IDi-5	Il soggetto non si confronta o si estranea rispetto ad aspetti contradditori di sé, ma solo quando le situazioni sono più confusive (o) soggettivamente minacciose	IDe-5	Il soggetto non si confronta o si estranea rispetto ad aspetti contradditori di altre persone, ma solo quando le situazioni sono più confusive (o) soggettivamente minacciose
IDi-6	Quando il soggetto si confronta con aspetti divergenti di sé, tende prioritariamente a non interrogarsi su come si sente (e/o) a svalutarsi (e/o) ad auto-affermarsi	IDe-6	Quando il soggetto si confronta con aspetti divergenti delle relazioni con gli altri, sente il bisogno di evitare il confronto (e/o) di cercare accettazione (e/o) di svalutare gli altri
IDi-7	Quando il soggetto si confronta con aspetti divergenti di sé, non ha bisogno di evitare il confronto con gli altri, (e/o) di adeguarsi per forza al loro consenso, (e/o) di svalutare le posizioni altrui	IDe-7	Quando il soggetto si confronta con aspetti divergenti delle relazioni con gli altri, non tende sistematicamente a preoccuparsi, (e/o) a svalutarsi, (e/o) ad auto-affermarsi

**Table 3:** Psychopathological Functioning Levels – Rating Scale (PFL-RS): Italian schedule

COMPRENSIONE			
	*Prospettiva interna*		*Prospettiva esterna*
COi-1	Il soggetto confonde uno o più vissuti personali con i dati oggettivi (e/o) riferisce a sé senza motivo eventi estranei (e/o) non rispetta le connessioni logiche del pensiero	COe-1	Le rappresentazioni soggettive di alcuni stati emotivi (e/o) comportamenti altrui sono disorganizzate sul piano logico (e/o) le evidenze critiche che ne invalidano i contenuti sono ignorate (e/o) rifiutate
COi-2	Il soggetto non è in grado di comprendere e descrivere il proprio attuale stato emotivo (e/o) comportamento, se non attraverso aspetti parziali e occasionali	COe-2	Il soggetto non è in grado di comprendere e descrivere gli stati emotivi (e/o) le motivazioni (e/o) del comportamento altrui, se non attraverso aspetti parziali e occasionali
COi-3	Il soggetto comprende il proprio stato emotivo (e/o) comportamento, ma non è capace di comprendere contemporaneamente quello degli altri	COe-3	Il soggetto comprende lo stato emotivo (e/o) il comportamento degli altri, ma non è capace di sentirsi contemporaneamente compreso da loro
COi-4	Il soggetto comprende solo razionalmente le situazioni, mentre le emozioni divergenti che possono nascere dal confronto non vengono integrate e vissute insieme	COe-4	Il soggetto mostra di rifugiarsi impropriamente in considerazioni concrete quando si tratta di comprendere gli aspetti contradditori di una situazione
COi-5	Il soggetto non coglie bene alcuni vissuti personali, limitati e specifici, perché ne annulla le sfumature delle caratteristiche (e/o) delle cause (e/o) degli effetti	COe-5	Il soggetto non coglie bene alcuni segnali ambientali, limitati e specifici, perché ne annulla le sfumature delle caratteristiche (e/o) delle cause (e/o) degli effetti
COi-6	Se insorgono difficoltà rispetto ai propri progetti, il soggetto ne sminuisce l’importanza (e/o) privilegia la ricerca del piacere o del predominio rispetto ad altri obiettivi (e/o) rinuncia a modificarli	COe-6	Se insorgono difficoltà rispetto ai propri progetti, il soggetto tende a rinunciarvi (e/o) privilegia l’approvazione rispetto a perseguire altri obiettivi (e/o) sopravvaluta o sottovaluta le condizioni di contesto
COi-7	Il soggetto è consapevole del valore dei propri progetti anche quando valuta gli eventuali aspetti sfavorevoli delle condizioni di contesto	COe-7	Se insorgono difficoltà relative ai propri progetti, il soggetto valuta l’opportunità di affrontarle o evitarle con realismo, senza eccessi di allarme o di sfiducia

**Table 4:** Psychopathological Functioning Levels – Rating Scale (PFL-RS): Italian schedule

EMOZIONI NEGATIVE			
	*Prospettiva interna*		*Prospettiva esterna*
EMi-1	In modo stabile (e/o) in talune situazioni, il soggetto manifesta emozioni negative o positive incongrue per qualità e intensità	EMe-1	In modo stabile (e/o) in talune situazioni, l’umore (e/o) le emozioni del soggetto appaiono immodificabili e incongrue in base alle circostanze
EMi-2	La minaccia di smarrire la propria individualità è intollerabile e produce, anche in risposta a stimoli neutri o positivi, sentimenti molto mutevoli di angoscia (e/o) di rabbia (e/o) di vuoto	EMe-2	La minaccia che la propria individualità non sia riconoscibile è intollerabile e produce, anche in risposta a stimoli neutri o positivi, sentimenti molto mutevoli di angoscia (e/o) di rabbia (e/o) di vuoto
EMi-3	La minaccia rispetto ad aspetti ambivalenti di sé è tollerabile solo occasionalmente e produce, senza modulazione, sentimenti di angoscia (e/o) di intolleranza (e/o) di vuoto	EMe-3	La minaccia rispetto ad aspetti ambivalenti di altri è tollerabile solo occasionalmente e produce, senza modulazione, sentimenti di angoscia (e/o) di intolleranza (e/o) di vuoto
EMi-4	L’angoscia rispetto ad aspetti ambivalenti di sé si manifesta con disconoscimenti oppositivi (e/o) sentimenti di vuoto; può essere espressa solo in contesti protetti	EMe-4	L’angoscia rispetto alla qualità contradditoria delle esperienze è colta solo in contesti protetti e si manifesta con disconoscimenti oppositivi (e/o) sentimenti di vuoto
EMi-5	Solo in situazioni confusive e minacciose l’angoscia rispetto ad aspetti ambivalenti di sé non è tollerata e produce rifiuti oppositivi (e/o) sentimenti di vuoto di intensità variabile	EMe-5	Solo in situazioni confuse e minacciose l’angoscia rispetto ad aspetti relazionali ambivalenti non è tollerata e produce rifiuti oppositivi (e/o) sentimenti di vuoto di intensità variabile
EMi-6	Di fronte all’eccessivo coinvolgimento in talune situazioni esistenziali, il soggetto non sa ridurre l’ansia (e/o) rimuove o reprime la rabbia (e/o) è insicuro di sé	EMe-6	Di fronte all’eccessivo coinvolgimento in talune situazioni esistenziali, il soggetto non sa ridurre l’ansia (e/o) rimuove o reprime la rabbia (e/o) è insicuro degli altri
EMi-7	Rispetto a possibili situazioni dolorose (e/o) stressanti, il soggetto è capace di ridurre convenientemente l’angoscia	EMe-7	L’attenzione del soggetto alla prevenzione del danno (e/o) alle modalità per gestirlo non limita in modo esagerato la percezione del benessere

**Table 5:** Psychopathological Functioning Levels – Rating Scale (PFL-RS): Italian schedule

AZIONE-REGOLAZIONE			
	*Prospettiva interna*		*Prospettiva esterna*
ARi-1	Il soggetto mostra comportamenti alienati (e/o) stereotipati (e/o)auto-distruttivi che derivano da un compromesso esame di realtà	ARe-1	Il soggetto mostra comportamenti alienanti (e/o) stereotipati (e/o) etero- distruttivi che derivano da un compromesso esame di realtà
ARi-2	Il soggetto pone in atto tentativi suicidari (e/o) comportamenti auto-lesivi (e/o) alienati che sostituiscono altri contenuti mentali ed espressioni di bisogno	ARe-2	Il soggetto pone in atto comportamenti etero-lesivi violenti (e/o) alienanti che sostituiscono altri contenuti mentali ed espressioni di bisogno
ARi-3	Il soggetto minaccia di agire comportamenti suicidari (e/o) auto-lesivi (e/o) alienati che sostituiscono altri contenuti mentali ed espressioni di bisogno	ARe-3	Il soggetto mostra comportamenti clastici (e/o) minaccia di agire comportamenti eterolesivi violenti (e/o) alienanti che sostituiscono altri contenuti mentali ed espressioni di bisogno
ARi-4	Il soggetto fantastica di porre in atto comportamenti suicidari (e/o) auto-lesivi (e/o) alienati che sostituiscono altri contenuti mentali ed espressioni di bisogno	ARe-4	Il soggetto fantastica di porre in atto comportamenti clastici (e/o) alienanti (e/o) etero-lesivi violenti che sostituiscono altri contenuti mentali ed espressioni di bisogno
ARi-5	In situazioni specifiche, il comportamento del soggetto non tiene conto del fatto che esistono discrepanze tra significati soggettivi e richieste ambientali	ARe-5	In qualche situazione il comportamento del soggetto è impulsivo (e/o) inibito perché non considera in modo realistico i fattori favorevoli (e/o) sfavorevoli
ARi-6	Il soggetto persegue i propri intenti realistici in modo eccessivamente ridotto e assume comportamenti evitanti (e/o) troppo rassegnati (e/o) troppo preoccupati	ARe-6	Le discrepanze tra propositi soggettivi e richieste ambientali producono comportamenti rinunciatari (e/o) troppo passivi (e/o) troppo preoccupati
ARi-7	Il soggetto persegue i propri intenti realistici, senza assumere comportamenti evitanti (e/o) rassegnati, ed è in grado di dilazionare il soddisfacimento dei propri bisogni	ARe-7	Le discrepanze tra propositi soggettivi e richieste ambientali non comportano comportamenti eccessivamente rinunciatari (e/o) passivi (e/o) testardi

**Table 6:** Psychopathological Functioning Levels – Rating Scale (PFL-RS): Italian schedule

RELAZIONI SOCIALI			
	*Prospettiva interna*		*Prospettiva esterna*
SOi-1	Il soggetto distorce senza possibilità di critica il significato delle situazioni, (e/o) di alcune di esse, (e/o) di alcune regole della convivenza sociale	SOe-1	Almeno un aspetto della convivenza sociale (e/o) delle relazioni del soggetto è stabilmente compromesso dal mancato esame di realtà
SOi-2	Una stabile capacità di soddisfacimento dei bisogni fondamentali (e/o) di relazione continuativa secondo le norme della convivenza civile risulta compromessa	SOe-2	Il soggetto alterna grandi richieste di presenza da parte degli altri e allontanamenti improvvisi (e/o) inaspettati
SOi-3	La solitudine è intollerabile e viene evitata anche a costo di immaginare relazioni che non possono alterarsi (e/o) terminare	SOe-3	Il distacco relazionale è vissuto come un abbandono a cui il soggetto attribuisce un valore enorme (e/o) nessun valore
SOi-4	Il soggetto persegue (e/o) evita gli impegni relazionali della vita affettiva (e/o) dello studio - lavoro (e/o) del tempo libero secondo motivazioni assolutizzate	SOe-4	I rapporti sociali che comportano intimità (e/o) forti emozioni contrastanti non sono sopportati con continuità (e/o) sono affrontati solo se il soggetto dipende da una guida esterna
SOi-5	La capacità del soggetto di essere autonomo e di impegnarsi nel confronto con gli altri è fonte di difficoltà, anche in assenza di eventi negativi	SOe-5	Il soggetto ha difficoltà ad alternare distanza e vicinanza relazionale (e/o) impegno e astensione (e/o) attivazione e richiesta d’aiuto (e/o) cooperazione e opposizione
SOi-6	Nell’instaurare o continuare relazioni significative, il soggetto mostra, in qualche ambito, un’eccessiva tendenza all’evitamento (e/o) al pessimismo (e/o) ad un’eccessiva tensione (e/o) passività	SOe-6	Il soggetto si prospetta relazioni significative ma, in caso di difficoltà, mostra un eccessivo aumento del timore di promuoverle (e/o) della preoccupazione di come possano procedere (e/o) della tendenza ad adeguarsi agli altri
SOi-7	Il soggetto si prospetta relazioni significative e affronta le difficoltà senza atteggiamenti troppo rinunciatari, (e/o) scoraggiati, (e/o) preoccupati	SOe-7	Il soggetto si mostra sufficientemente sereno e fiducioso nell’instaurare (e/o) continuare relazioni significative

**Table 7:** Psychopathological Functioning Levels – Rating Scale (PFL-RS): English schedule

IDENTITY			
	*Internal perspective*		*External perspective*
IDi-1	The individual claims to perceive the co-presence of other entities into himself/herself	IDe-1	The individual is persuaded that, in certain cases, people have the power to expropriate others of their inner identity (and/or) freedom
IDi-2	The self-representation of the individual corresponds to some temporary attitudes of himself/herself without being critically aware of other opposite attitudes	IDe-2	The subjective representations of others’ behaviors seem to be imposed by an occasional urge and are disconnected from those in the past
IDi-3	In order to be self-represented as a whole the individual adopts a sectorial image of himself/herself and such an image is experienced as completely positive (or) negative	IDe-3	In order to make himself/herself an idea of the others (and/or) of the relationships the individual uses a sectorial image which is completely positive (or) negative
IDi-4	When facing his/her contradictory aspects the individual minimizes (or) disawovs their relevance	IDe-4	When facing contradictory meanings of social situations the individual minimizes (or) disawovs their relevance
IDi-5	The individual does not face the contradictory aspects of himself/herself or becomes estranged, but only when the situations are confused (or) subjectively perceived as threatening	IDe-5	The individual does not face the contradictory aspects of the others or becomes estranged, but only when the situations are confused (or) subjectively perceived as threatening
IDi-6	When the individual faces conflicting aspects of himself/herself primarily tends not to ask himself/herself about his/her feelings (and/or) to devalue himself/herself (and/or) to self-assert himself/herself	IDe-6	When the individual faces conflicting aspects of the relationships with others needs to avoid the comparison (and/or) look for acceptance (and/or) devalue others
IDi-7	When the individual faces conflicting aspects of himself/herself there is no need to avoid confrontation with others (and/or) adapt to others’ approval (and/or) devalue other’s opinions	IDe-7	When the individual faces conflicting aspects of the relationships with others he/she does not tend systematically to worry (and/or) devalue himself/herself (and/or) self-assert himself/herself

**Table 8:** Psychopathological Functioning Levels – Rating Scale (PFL-RS): English schedule

COMPREHENSION			
	*Internal perspective*		*External perspective*
COi-1	The individual confuses one or more personal life experiences with objective data (and/or) refers external events to himself/herself with no reason (and/or) does not follow logical thinking	COe-1	The subjective representations of others’ certain emotional states (and/or) behaviors are disorganized from a logical standpoint (and/or) critical evidence against such representations or behaviors are ignored (and/or) refused
COi-2	The individual cannot understand and describe his/her current emotional state (and/or) behavior but through partial and occasional aspects	COe-2	The individual cannot understand and describe others’ emotional states (and/or) the motivation of others’ behavior but through partial and occasional aspects
COi-3	The individual understands his/her current emotional state (and/or) behavior but he/she cannot understand at the same time those of others	COe-3	The individual understands others’ current emotional state (and/or) behavior but he/she cannot feel at the same time understood by others
COi-4	The individual understands only rationally the situations while conflicting emotions possibly arising from the confrontation are not integrated and perceived as a whole	COe-4	The individual acts as improperly taking shelter in concrete considerations when coping with the understanding of the conflicting aspects of a certain situation
COi-5	The individual does not understand well some defined and specific personal life experiences because he/she erases the different shades of the characteristics (and/or) causes (and/or) effects	COe-5	The individual does not understand well certain defined and specific signals in the environment because he/she erases the different shades of the characteristics (and/or) causes (and(or) effects
COi-6	If difficulties concerning his/her projects arise the individual diminishes their relevance (and/or) prefers to seek pleasure or predominance of other objectives (and/or) renounces to modify them	COe-6	If difficulties concerning his/her projects arise the individual tends to give up (and/or) to endorse the approval rather than pursue other objectives (and/or) to overestimate or underestimate the environmental conditions
COi-7	The individual is aware of the value of his/her projects even when evaluating the eventual negative aspects of the environmental conditions	COe-7	If difficulties concerning his/her projects arise the individual evaluates the opportunity to face or avoid them with realism and without excessive apprehension or distrust

**Table 9:** Psychopathological Functioning Levels – Rating Scale (PFL-RS): English schedule

NEGATIVE EMOTIONS			
	*Internal perspective*		*External perspectivehe*
NEi-1	Permanently (and/or) in certain situations the individual shows negative or positive emotions which are incongruous for quality and intensity	NEe-1	Permanently (and/or) in certain situations the individual’ mood (and/or) emotions seem unmodifiable and incongruous for the circumstances
NEi-2	The threat to lose his/her individuality is intolerable and generates, even when responding to neutral or positive stimuli, very unstable feelings of distress (and/or) anger (and/or) emptiness	NEe-2	The threat that his/her individuality is not recognizable is intolerable and generates, even when responding to neutral or positive stimuli, very unstable feelings of distress (and/or) anger (and/or) emptiness
NEi-3	The threat represented by his/her own ambivalent aspects is only occasionally tolerable and generates, without modulation, feelings of distress (and/or) anger (and/or) emptiness	NEe-3	The threat represented by others’ ambivalent aspects is only occasionally tolerable, and generates, without modulation, feelings of distress (and/or) anger (and/or) emptiness
NEi-4	The distress produced by his/her own ambivalent aspects is shown as oppositional disavowals (and/or) feelings of emptiness; such a distress can be expressed only in a safe environment	NEe-4	The distress produced by the contradictory quality of his/her own experiences is grasped only in a safe environment and is shown as oppositional disavowals (and/or) feelings of emptiness
NEi-5	Only in confused and threatening situations the distress produced by the individual’s ambivalent aspects is not tolerated and generates oppositional disavowals (and/or) different degrees of feelings of emptiness	NEe-5	Only in confused and threatening situations produced by ambivalent relational experiences is not tolerated generates oppositional disavowals (and/or) different degrees of feelings of emptiness
NEi-6	When facing an excessive involvement in certain existential situations the individual cannot reduce anxiety (and/or) restrains or represses anger (and/or) is insecure of his/herself	NEe-6	When facing an excessive involvement in certain existential situations the individual cannot reduce anxiety (and/or) restrains or represses anger (and/or) is insecure of others
NEi-7	With respect to possible painful (and/or) stressful situations the individual can reduce conveniently his/her distress	NEe-7	The attention the individual pays to preventing harm (and/or) to the modalities to cope with it does not limit the perception of his/her wellbeing

**Table 10:** Psychopathological Functioning Levels – Rating Scale (PFL-RS): English schedule

ACTION- REGULATION			
	*Internal perspective*		*External perspective*
ARi-1	The individual shows alienated (and/or) stereotyped (and/or) self-destructive behaviors deriving from an impaired reality testing	ARe-1	The individual shows alienated (and/or) stereotyped (and/or) destructive other-behaviors deriving from an impaired reality testing
ARi-2	The individual commits suicidal attempts (and/or) self-harm (and/or) alienated behaviors that substitute other mental contents and expressions of needs	ARe-2	The individual commits alienated (and/or) aggressive behaviors towards others that substitute other mental contents and expressions of needs
ARi-3	The individual threatens to commit suicidal attempts (and/or) self-harm (and/or) alienated behaviors that substitute other mental contents and expressions of needs	ARe-3	The individual shows clastic behaviors (and/or) threatens to commit alienated (and/or) aggressive behaviors towards others that substitute other mental contents and expressions of needs
ARi-4	The individual daydreams to commit suicidal attempts (and/or) self-harm (and/or) alienated behaviors that substitute other mental contents and expressions of needs	ARe-4	The individual daydreams to commit clastic (and/or) alienated (and/or) aggressive behaviors towards others that substitute other mental contents and expressions of needs
ARi-5	In specific situations the individual’s behavior does not consider the existing discrepancy between subjective meanings and environmental requests	ARe-5	In certain situations the individual’s behavior is impulsive (and/or) inhibited because he/she does not consider in a realistic way the favorable (and/or) unfavorable factors
ARi-6	The individual pursues his/her own realistic aims in an excessively reduced way and assumes avoidant (and/or) too resigned (and/or) too worried behaviors	ARe-6	The discrepancies between subjective intentions and environmental requests produce defeatist (and/or) too passive (and/or) too preoccupied behaviors
ARi-7	The individual pursues his/her own realistic aims without assuming avoidant (and/or) resigned behaviors and he/she can postpone satisfaction of his/her own needs	ARe-7	The discrepancies between subjective intentions and environmental requests do not produce excessively defeatist (and/or) passive (and/or) stubborn behaviors

**Table 11:** Psychopathological Functioning Levels – Rating Scale (PFL-RS): English schedule

SOCIAL SKILLS			
	*Internal perspective*		*External perspective*
SSi-1	The individual uncritically distorts the meaning of the situations (and/or) of some of them (and/or) some rules of social living	SSe-1	At least one aspect of social living (and/or) individual’s relationships is permanently impaired by an altered reality testing
SSi-2	A stable ability to satisfy basic needs (and/or) the needs of a stable relationship according to the rules of social living is impaired	SSe-2	The individual alternates a great demand for the presence of others and sudden (and/or) unexpected estrangement
SSi-3	Loneliness is intolerable and is avoided even imagining relationships that cannot change (and/or) end	SSe-3	The relational detachment is perceived as an abandonment which is hugely (and/or) not valued
SSi-4	The individual pursues (and/or) avoids the relational commitments of affective life (and/or) of study/work (and/or) spare time according to absolutized motivations	SSe-4	The social relationships that entail intimacy (and/or) strong contrasting emotions are not supported uninterruptedly (and/or) are handled only depending on an external guidance
SSi-5	The individual’s ability to be autonomous and to engage in a comparison with others is a source of difficulty, even in the absence of negative events	SSe-5	The individual has difficulties in alternating relational distance and closeness (and/or) effort and self-restraint (and/or) activation and asking for help (and/or) cooperation and opposition
SSi-6	When establishing or continuing significant relationships the individual shows in some aspects an excessive tendency to avoidance (and/or) pessimism (and/or) excessive tension (and/or) passivity	SSe-6	The individual imagines significant relationships but in case of need shows an excessive increase in the fear of promoting them (and/or) in the preoccupation about how they may proceed (and/or) in the tendency to adapt himself/herself to others
SSi-7	The individual imagines significant relationships and copes with the difficulties without too defeatist (and/or) discouraged (and/or) preoccupied attitudes	SSe-7	The individual appears sufficiently calm and trustful in the establishment (and/or) continuation of meaningful relationships

PFL-RS can be repeated multiple times to assess patient’s psychopathological dysfunctions during the course of treatments.

Raters are asked to choose the definition that best describes patient’s worst psychic functioning at that moment. PFL rating may be different in each area of the same patient. Although short, a specific training is recommended in order to provide guidelines to conduct this assessment.

In summary, assessment procedures include a semi-structured interview (lasting approximately 1 h) and subsequent open interviews (lasting approximately 30 min), containing specific anamnestic questions and referring to clinical criteria.

More in detail, the interview is focused on recognizing different phases [2]: 1. Opening (symptoms, life events). 2. Dysfunctional repetitive patterns. 3. Self and others perception in different areas. 4. Past and present life style and life tasks. Unconscious processes can be inferred by contradictions and narrative structure.

Raters are also required to mark down the quality of patient-therapist relationship; this will be considered as a control variable during the evaluation, eventually suggesting absence of relationship, opposition versus willingness, idealized, dependent, supportive, fearful, preoccupate, dismissing, or cooperative relationship, only to name a few (see Tables 12 and 13).

**Table 12:** According to the Psychopathological Functioning Levels – Rating Scale (PFL-RS) raters are required to mark down the quality of patient-therapist relationship. Italian schedule

QUALITA’ DELLA RELAZIONE TERAPEUTICA
T-NO: ASSENZA DI RELAZIONE TERAPEUTICA
Non è in atto alcun trattamento oppure si è interrotto.
T-OD: ALTERNANZA DI MANIFESTAZIONI DI OPPOSITIVITA’ E DISPONIBILITA’ AL TERAPEUTA
Il terapeuta non è vissuto nella sua interezza, ma per alcune funzioni parziali che il paziente immagina possa svolgere: fargli da specchio, ospitare debolezze o minacce, essere presente senza relazionarsi.
*Come si manifesta*: nei casi più gravi consiste nel chiedere una immediata disponibilità, cui segue la fuga quando il terapeuta può essere presente, oppure comporta un’alternanza tra grande soddisfazione se il terapeuta risponde alle funzioni che il paziente gli ha assegnato e grande oppositività e delusione se il terapeuta non soddisfa le attese.
T-ID: RELAZIONE IDEALIZZATA COL TERAPEUTA
Il rapporto più stabile del paziente è quello col terapeuta, immaginato come sempre comprensibile in quanto interamente positiva e senza rischi di abbandono.
*Come si manifesta*: si riconosce perché il paziente mostra di non saper fare a meno del terapeuta, deve continuamente verificarne la presenza, tollera deleghe all’accoglimento solo in favore di persone vissute come strettamente in sintonia con il terapeuta.
T-DI: RELAZIONE DIPENDENTE COL TERAPEUTA
Il paziente vive il terapeuta in modo realistico, ma nell’ambito di un rapporto immaginato come assolutamente privilegiato e stabile.
*Come si manifesta*: si riconosce perché il paziente mostra di non saper fare a meno del terapeuta, anche se accetta la sua assenza, tollera deleghe all’accoglimento solo con persone che non siano vissute in antitesi al terapeuta.
T-SU: RELAZIONE TERAPEUTICA CON ECCESSIVO BISOGNO DI SOSTEGNO
Si tratta di una relazione tra due persone distinte, ma il soggetto teme che il legame con il terapeuta sia debole e fragile.
*Come si manifesta*: la relazione cooperativa di sostegno si riconosce dal fatto che il paziente si pone in atteggiamento prevalente di bisogno (bimbo, scolaro, sofferente).
T-AL: RELAZIONE TERAPEUTICA DIALOGICA, CON ECCESSIVO ALLARME
Si tratta di relazioni tra due persone distinte, ma il soggetto teme il legame con un terapeuta poco affidabile
*Come si manifesta*: la relazione dialogica è sufficientemente paritaria, pur nelle diversità, ma per essere accettato, il p. evita di dare troppa importanza alle posizioni del terapeuta.
T-RC: RELAZIONE TERAPEUTICA DIALOGICA, CON ECCESSIVA RICERCA DI RICONOSCIMENTO
Si tratta di relazioni tra due persone distinte, ma il soggetto teme il legame con un terapeuta troppo lontano.
*Come si manifesta*: la relazione dialogica è sufficientemente paritaria, pur nelle diversità, ma per essere accettato, il p. dà troppa importanza alle posizioni del terapeuta.
T-AS: RELAZIONE TERAPEUTICA DIALOGICA, CON ECCESSIVI BISOGNI DI ASSERTIVITA’
Si tratta di una relazione tra due persone distinte, ma il paziente teme che il legame con il terapeuta sia molto competitivo e impegnativo.
*Come si manifesta*: la relazione dialogica è sufficientemente paritaria, pur nelle diversità, ma per potersi affermare, il paziente riduce l’importanza delle posizioni del terapeuta.
T-CO: RELAZIONE TERAPEUTICA DIALOGICA COOPERATIVA
Si tratta di una relazione tra due persone distinte e cooperanti nella concordia e nel confronto.
*Come si manifesta*: la relazione dialogica è sufficientemente paritaria, pur nelle diversità, e il paziente è disponibile a considerare le posizioni del terapeuta.

**Table 13:** According to the Psychopathological Functioning Levels – Rating Scale (PFL-RS) raters are required to mark down the quality of patient-therapist relationship. English schedule

QUALITY OF THE THERAPEUTIC RELATIONSHIP
T-NO: ABSENCE OF THE THERAPEUTIC RELATIONSHIP
No current treatments or the treatment has been discontinued.
T-OD: ALTERNATION OF DEMONSTRATION OF OPPOSITIONAL BEHAVIORS AND OPENNES TOWARDS THE THERAPIST
The therapist is not considered as a whole but only concerning some partial functions as imagined by the patient: mirroring, accepting weaknesses or threats, being present without a relationship.
*How it can be shown:* in most severe cases this consists in asking an immediate availability and then run away when the therapist can be present or as an alternation between great satisfaction when the therapist responds to the functions that the patient assigned to him/her and great oppositional behaviors when the therapist does not fulfill the patient’s expectations.
T-ID: IDEALIZED RELATIONSHIP WITH THE THERAPIST
The most stable relationship of the patient is with his/her therapist who is imagined as always comprehensible since completely positive and not entailing any risks of abandonment.
*How it can be shown:* it is recognizable because the patient seems to be unable to do without the therapist and the patient has to continuously verify the therapist’s presence and tolerates exceptions to being accepted only in favor of people who are perceived as in strict harmony with the therapist.
T-DI: DEPENDENT RELATIONSHIP WITH THE THERAPIST
The patient lives the therapist in a realistic way but in the context of a relationship which is imagined as absolutely privileged and stable.
*How it can be shown*: it is recognizable because the patient seems to be unable to do without the therapist although the therapist’s absence is tolerated, and exceptions to being accepted are tolerated only favor of people who are perceived as not in contrast with the therapist.
T-SU: THERAPEUTIC RELATIONSHIP WITH EXCESSIVE NEED OF SUPPORT
It is a relationship between two distinct persons but the individual is afraid that the relationship with the therapist is weak and fragile.
*How it can be shown*: the supportive relationship is recognizable because the patient is mainly characterized by an attitude of need (e.g., child, schoolchildren, sufferer).
T-AL: DIALOGIC THERAPEUTIC RELATIONSHIP, WITH EXCESSIVE APPREHENSION
It is a relationship between two different persons but the individual is afraid of the relationship with a poorly reliable therapist.
*How it can be shown*: the dialogic relationship is sufficiently dialogic but, in order to be accepted, the patient avoids to value the therapist’s positions.
T-RC: DIALOGIC THERAPEUTIC RELATIONSHIP, WITH EXCESSIVE NEED FOR REWARD
It is a relationship between two different persons but the individual is afraid of the relationship with a therapist who is too far.
*How it can be shown*: the relationship is sufficiently dialogic but, in order to be accepted, the patient extremely values the therapist’s positions.
T-AS: DIALOGIC THERAPEUTIC RELATIONSHIP, WITH EXCESSIVE NEED FOR ASSERTIVENESS
It is a relationship between two different persons but the individual is afraid that the relationship with the therapist is very competitive and demanding.
*How it can be shown*: the relationship is sufficiently dialogic but, in order to affirm himself/herself, the patient reduces the importance of the therapists’ positions.
T-CO: COOPERATIVE DIALOGIC THERAPEUTIC RELATIONSHIP
It is a relationship between two different persons who cooperate with understanding and confrontation.
*How this can be shown*: the relationship is sufficiently dialogic and the patient is open to consider the therapist’s positions.

### (Step J) Preliminary data on the administration of the PPFLs, PFLs and PFL-RS to different clinical samples

In order to assess patients’ dysfunctions, more than a decade ago, the clinicians of the Mental Health Department of the ASL Turin 4 in Italy started using the “Primary Psychotherapeutic Focus Levels” (PPFLs) and “Personality Functioning Levels” (PFLs), two sets of definitions pioneering the PFL-RS (see Step G).

More in detail, from 2004 to 2007, when conducting the Brief-Adlerian Psychodynamic Psychotherapy (B-APP) therapists based their treatment strategies on the description of patient’s psychological functioning according to the PPFLs. Such a psychopathology-based psychotherapy showed efficacy and improved symptoms and global functioning in a sample of patients with Generalized Anxiety Disorder [83].

Furthermore, from 2006 to 2011, the multidisciplinary therapeutic team of Mental Health Service (MHS) in Chivasso (Turin, Italy) was trained and supervised to assess different PFLs which served as benchmark for the Supervised Team Management (STM) of clinical projects for patients with Borderline Personality Disorder in a community setting. Treatments included medications, unstructured psychological support (UPS) focused on socio-relational impairment or a specific time-limited psychotherapy (Sequential Brief-Adlerian Psychodynamic Psychotherapy – SB-APP), and rehabilitative interventions [22, 80]. A set of definitions of different impairment levels concerning the same five core areas that will be envisaged to be investigated by PFL-RS (see Table 14) was evaluated by two independent raters, in order to adjust the modulations of the therapeutic relationship to fit each specific patient [22, 23].

**Table 14:** Main psychopathological items in the description of different Psychopathological Functioning Levels (PFLs) in Borderline Personality Disorder

Items	PFL 1	PFL 2	PFL 3	PFL 4
ID	Partial symbolic and pre-symbolic representations of self (nuclear identity)	Splitting and idealization of self and others representations (split identity)	Avoiding consequences of being aware of one’s own and others contradictory qualities (anti-ambivalent identity)	Anti-ambivalent and hyper-ambivalent aspects of identity
CO	Impaired comprehension of one’s own and others behaviors in terms of thoughts, desires and expectations	Comprehension of one’s own and others behaviors, thoughts and emotions, only if they do not upset self-image	Concrete thought	Poor tolerance of contradictory aspects of one’s own and others behaviors, thoughts and emotions
			When divergent motivations stem from comprehension of one’s own and others behaviors, thoughts and emotions, they are not integrated	
NE	Anger, depression, feelings of emptiness	Irritation, depression, feelings of emptiness	Anger recognition, shame, depression, feeling of emptiness	Guilt, sadness, dissatisfaction, feelings of emptiness
AR	Self-damaging and/or alienating behaviors	Threats of self-harming and/or alienating behaviors	Ideas of self-harming and/or alienating behaviors	At some extent, impulsive and/or blocked behaviors
SS	Poor capability to manage social autonomies	Unstable tolerance for engagements and relations	Attempts to work	Poor flexibility in distancing or approaching others
			Low tolerance of loneliness	

Legend:

*PFL* Psychopathological Functioning Level, *ID* Identity, *CO* Comprehension, *NE* Negative Emotions, *AR* Action Regulation, *SS* Social Skills

Modified from Ferrero A.: The Model of Sequential Brief-Adlerian Psychodynamic Psychotherapy (SB-APP): Specific Features in the Treatment of Borderline Personality Disorder. Research in Psychotherapy: Psychopathology, Process and Outcome, 2012, Vol. 15, No. 1, 32–45

Overall, STM had to consider that patients, when at PFL-1 and PFL-2, unconsciously fear that their fragile identity might collapse (ID 1-2). In this regard, empathic validation and, to a lesser degree, clarification and affirmation could effectively convey constructive experiences opposite to precarious self-cohesion (ID 1-2). The latter is represented by patient’s inability to think when others are present (CO 1-2) and to tolerate (NE 1-2) the therapist as endowed of a separate existence (SS 1-2). At PFL-3, STM took into account that patients are largely engaged in denying self-other contradictory images (ID 3) as relevant for their life and behaviors (AR 3-4), since at PFL-4 this attitude only appears when facing specific tearing emotions and situations (ID 4). Treatments were aimed at reducing the sense of emptiness (NE 3-4; SS 3-4) and increasing continuity and adaptation: systematic consideration was paid to patients’ concrete way of thinking (CO 3-4), acting-out (AR 3-4), and discontinuities in their social relationships involving intimacy (SS 3-4).

In a preliminary clinical randomized study, thirty-five outpatients with BPD meeting inclusion criteria of service heavy users were evaluated. The study was aimed to evaluate the efficacy of PFLs informed clinical projects (STM), both including UPS and SB-APP, compared to Treatment As Usual (TAU) [80].

PFLs’ scoring was not included as patients’ assessment measure at baseline, since PFLs’ set of definitions had not been validated yet. However, it was found that overall PFL’s scoring distribution at baseline varied depending on each considered psychopathological area (see Table 15) and it did not overlap with the scores on Clinical Global Impression (CGI) [84], Symptom Checklist-90 Revised (SCL-90-R) [85] and Global Assessment of Functioning (GAF) [69], both in UPS and SB-APP group, thus providing specific additional clinical information (see Table 15). Compared to Treatment As Usual (TAU), after one year of treatment, STM showed an overall improvement in clinical severity (CGI) [84], global functioning (GAF) [69], and all nine psychopathological domains included in the diagnosis of BPD (CGI-M items 1–9) [80]. Both compliance to treatments and quality of therapeutic alliance were greater as well.

**Table 15:** Baseline clinical features of patients with Borderline Personality Disorder receiving unstructured psychological support and Sequential Brief-Adlerian Psychodynamic Psychotherapy arms of treatment

		UPS	SB-APP
	*N* (/%)	Mean (SD)	Mean (SD)
ID		2.88 (1.05)	2.22 (0.94)
1	7 (20)		
2	9 (25.7)		
3	12 (34.3)		
4	7 (20)		
CO		2.88 (1.11)	2.27 (0.82)
1	6 (17.1)		
2	10 (28.6)		
3	12 (34.3)		
4	7 (20)		
NE		2.64 (0.78)	2.38 (0.5)
1	1 (2.9)		
2	17 (48.6)		
3	15 (42.9)		
4	2 (5.7)		
AR		3.58 (0.51)	3.11 (1.1)
1	3 (8.7)		
2	0 (0)		
3	14 (40)		
4	18 (51.5)		
SS		2.52 (0.71)	2.11 (0.83)
1	5 (14.3)		
2	16 (45.7)		
3	12 (34.3)		
4	2 (5.7)		
CGI Item1		3.8 (0.8)	4.3 (1)
SCL-90-R tot		130.9 (85.2)	156.6 (70)
GAF		57.4 (9.9)	60.2 (9.1)

Legend:

*UPS* unstructured psychological support, *SB-APP* Sequential Brief-Adlerian Psychodynamic Psychotherapy, *PFL* Psychopathological Functioning Level, *ID* Identity, *CO* Comprehension, *NE* Negative Emotions, *AR* Action Regulation, *SS* Social Skills, *CGI* Clinical Global Impression, *SCL-90-R* Symptom Checklist-90-R, *GAF* Global Assessment of Functioning

Modified from Amianto et al.: Supervised team management, with or without structured psychotherapy, in heavy users of a mental health service with borderline personality disorder: a two-year follow-up preliminary randomized study. BMC Psychiatry 2011 11:181

More in detail, one large (and also statistically significant) effect size was found for the outcome of identity disturbance (SMD 0.88, 95 % CI 0.16 to 1.60), a core dysfunctional area assessed by previous research [86].

This area was negatively correlated with self-harm and hospitalization rates. The reduction of dramatic occurrences and unscheduled interventions could highlight that a significant component of symptoms might be reduced by treatments informed by a specific assessment of underlying psychopathological dysfunctional areas [80].

Finally, the overall improvement in global functioning (GAF) [69] seems particularly important because patients’ quality of life remained poor, even after well-designed therapeutic interventions [80].

To date, PLS-RS was randomly administered to a small sample of 54 outpatients consecutively recruited at the Psychotherapy Unit in Settimo Torinese, Turin, Italy, together with the Temperament and Character Inventory (TCI) [37], Spielberger State and Trait Anxiety Inventory [87], Beck Depression Inventory [88], and the State-Trait Anger Expression Inventory [89]. Sixty % of the sample was composed by males and 40 % by females and their mean age was 29.03 ± 13.2 years (range: 17–64 years). The interviewers were either male (A.F.) or female researchers (S.F., E.C., B.S.) with MD or PhD credentials and extensive training (i.e., at least 5 years) in research. At the time of the study they were all working at the same facility (i.e., Psychotherapy Unit in Settimo Torinese, Turin, Italy) and all interviews were conducted there. Before starting this study, the researchers did not know the potential participants and contacted them using a face-to-face approach. Eight people refused to take part in this study because of lack of time. Before starting the interview, all participants were informed about the main goals of the study with the interviewers clarifying the main assumptions of this work. During the interview, only the researcher and the participant were present. Audio recording was not used because of research feasibility reasons; however the interviewer took notes of the interview during the assessment. Transcripts were not shared with participants and their feedback on the interview was encouraged but not formally required.

Three data coders coded the data according to the coding scheme provided by the authors. The Statistical Package for Social Sciences 21.0 (SPSS, SPSS Inc., Chicago, IL) was used for analysing the data. Concerning internal consistency, all PFL-RS subscales correlate strongly to each other. For example, Pearson’s correlation coefficient between internal and external perspectives of ID is *r* = .904, of CO is *r* = .901, NE is *r* = .922, AR is *r* = .614, SS is *r* = .,892. Moreover, HA on the TCI correlates positively with ID, CO, NE, and AR; finally, correlations have been also found between PFL-RS subscales and measures of depressive symptoms, anxiety, and anger. With more detail, ARe was inversely correlated with BDI (*r* = −.319, *p* = .024), STAI-trait (*r* = −.296, *p* = .035), and STAXI (*r* = −.396, *p* = .004). Also ARi was negatively correlated with STAI-trait (*r* = −.457, *p* = .001) and STAXI (*r* = −.431, *p* = .002). IDe was found to negatively correlate with STAXI (*r* = −.309, *p* = .029) as well as NE e (*r* = −.291, *p* = .040).

Finally, given the clinical use of this instrument considering each psychopathological areas and its global dimension, Cronbach’s alpha was .971. However, exhaustive research in order to assess PFL-RS validity, sensitivity and reliability is currently needed.

## Discussion

The comprehensive approach of this research aimed to ascertain to what extent psychopathological dysfunctions underlying mental disorders can be recognized considering seven different levels of PO [90], resulting from patients’ attempts to cope with suffering caused by vulnerabilities and life events.

PO cannot be directly assessed [12] and its definition was conveniently obtained by considering five primary PO manifestations that have been recognized as crucial.

Some instruments assessing PO use self-report questionnaires. Such an approach jeopardizes the ability to capture psychopathological dysfunctions since self-report tools are insufficiently tailored to describe mental contents and processes that are warded off through defences and self-serving biases [12]. Rating scales can be useful for standardized and comparable observations along with statistical analysis, although they are less detailed when compared to intensive research conducted on small samples or single cases [91].

PFL-RS will be first tested in Italy but it is likely that it will be used in different Countries since differences in personality patterns among individuals with mental disorders do not vary widely across cultures [92].

Although empirical foundations of psychodynamic-oriented classification of mental disorders is still far from being conclusive [93], preliminary findings suggest that PFL-RS could provide clinicians with a way to better understand specific aspects of patients’ psychopathological dysfunctions, using detailed features in a convenient way and without receiving extensive training.

Furthermore, according to previous research (PPFLs, PFLs), PFL-RS seems promising to inform in a specific way treatments strategies and goals, specifically concerning psychotherapy [22, 23, 90]; in fact, literature highlights that level of PO may interact with the type of treatment interventions [12].

## Conclusions

Guidelines for treatments can only rarely be obtained by a mere symptoms description of patients’ clinical condition and history. Different forms of psychopathology are underpinned by certain core disturbances which can relate to psychopathological dysfunctions with the latter being particularly helpful to guide treatments. However, it is not easy to identify such psychopathological dysfunctions; to date, some tools are available although highly complex and time consuming. To bridge this gap, with this study we aimed to develop the Psychopathological Functioning Levels – Rating Scale (PFL-RS), an easy-to-administer instrument focused on the identification of different levels of impairment in patients’ psychopathological functioning. This instrument investigates five dysfunctional areas: Identity (ID); Comprehension (CO); Negative Emotions (NE); Action-Regulation (AR); Social Skills (SS) providing 7 levels of severity for each area. The preliminary sample considered showed some limitations including a broad age range; however, further research is needed to perform a full validation of this tool. Notwithstanding, this instrument showed encouraging results with respect to the plan of treatment strategies although further research is warranted to confirm its psychometric properties and clinical adaptability.

## Abbreviations

APPs – TR, Adlerian Psychodynamic Psychotherapies - Training and Research; AR, Action-Regulation; CGI, Clinical Global Impression; CO, comprehension; DMRS, Defence Mechanisms Rating Scale; DSM, Diagnostic and Statistical Manual of Mental Disorders; EtSC, Etiopathogenetic Selection Criteria; FFM, Five Factor Model; GAF, Global Assessment of Functioning; ID, identity; NE, Negative Emotions; OPD-2, Operationalized Psychodynamic Diagnostics – 2; PDM, Psychodynamic Diagnostic Manual; PFL-RS, Psychopathological Functioning Levels – Rating Scale; PFLs, Personality Functioning Levels; PhSC, Phenomenological Selection Criteria; PO, personality organization; PPFLs, Primary Psychotherapeutic Focus Levels; QRC, Qualitative Research Criteria; SB-APP, Sequential Brief-Adlerian Psychodynamic Psychotherapy; SCL-90-R, Symptom Checklist-90 Revised; SS, Social Skills; STIPO, Structured Interview of Personality Organization; SWAP-200, Shedler Westen Assessment Procedure – 200; TCI, Temperament and Character Inventory; UPS, unstructured psychological support; VEP-PM, Vulnerability Events Personality - Psychopathological Model.
